# Mechanisms of Resistance to Hsp90 Inhibitor Drugs: A Complex Mosaic Emerges

**DOI:** 10.3390/ph4111400

**Published:** 2011-10-25

**Authors:** Peter W. Piper, Stefan H. Millson

**Affiliations:** Department of Molecular Biology and Biotechnology, University of Sheffield, Firth Court, Western Bank, Sheffield S10 2TN, UK; E-Mail: s.millson@sheffield.ac.uk

**Keywords:** Hsp90 inhibitors, cancer drug resistance, heat shock response, apoptosis

## Abstract

The molecular chaperone Hsp90 holds great promise as a cancer drug target, despite some of the initial clinical trials of Hsp90 inhibitor drugs having not lived up to expectation. Effective use of these drugs will benefit greatly from a much more detailed understanding of the factors that contribute to resistance, whether intrinsic or acquired. We review how cell culture studies have revealed a number of different mechanisms whereby cells can be rendered less susceptible to the effects of Hsp90 inhibitor treatment. A major influence is Hsp90 inhibition causing strong induction of the heat shock response, a stress response that increases cellular levels of prosurvival chaperones such as Hsp27 and Hsp70. Another problem seems to be that these inhibitors do not always access the Hsp90 proteins of the mitochondrion, forms of Hsp90 that—in cancer cells—are operating to suppress apoptosis. It should be possible to overcome these drawbacks through the appropriate drug redesign or with the combinatorial use of an Hsp90 inhibitor with a drug that targets either heat shock factor or the chaperone Hsp70. Still though, cells will often differ in the key antiapoptotic versus proapoptotic activities that are dependent on Hsp90, in the key steps in their apoptotic pathways responsive to Hsp90 inhibition or Hsp70 level, as well as the extents to which their survival is dependent on oncogenic tyrosine kinases that are clients of Hsp90. A systems approach will therefore often be required in order to establish the most prominent effects of Hsp90 inhibition in each type of cancer cell.

## 1. Introduction

Cancer progression is highly dependent on Hsp90, a molecular chaperone required for the activity of many of the oncoproteins that drive the growth, proliferation and survival of cancer cells. Not only is Hsp90 inhibited with a very high degree of selectivity using drugs that bind within its ADP/ATP binding pocket, but transformed cells are generally much more sensitive than normal cells to these inhibitors [1-3]. Several oncoproteins are inactivated and destabilized simultaneously in cancer cells treated with Hsp90 drugs, potentially allowing a combinatorial depletion of many cancer-causing pathways and a modulation of all of the hallmark traits of malignancy [4,5]. Hsp90 would appear therefore to represent the almost ideal cancer drug target.

To date the clinical trials of Hsp90 inhibitor drugs have been of variable outcome. Whilst promising activity has been shown against certain malignancies, certain other trials have proved disappointing [6-9]. Designing effective therapies around these drugs will entail a proper understanding of the molecular mechanisms whereby tumours are not always responsive to treatment. Many aspects have to be considered; such as differences in drug action on the various isoforms or conformations of Hsp90, dissimilarities in drug metabolism, the effects of Hsp90 inhibition on both normal and tumor cell physiology, the ability to cause either cell stasis or apoptosis, or an inability to achieve an effective level of drug in the tumour. Here we collate the evidence from cell culture studies on the diverse mechanisms of intrinsic and acquired resistance to Hsp90 drugs. First we discuss whether appreciable resistance might be possible through the mutation or altered covalent modification of the drug target—Hsp90 itself. Next, we consider the diverse cellular factors that can often influence how effectively growth is arrested, or apoptosis induced, in response to Hsp90 inhibitor treatment.

The Hsp90 drugs in clinical trials all act by binding within the ADP/ATP binding pocket of the Hsp90 N-terminal domain. These “N-domain inhibitors” exhibit two modes of binding within this pocket, exemplified by the interactions of two natural antibiotics; geldanamycin (GdA—a benzoquinone ansamycin produced by *Streptomyces hygroscopicus*) and radicicol (RAD—a fungal polyketide). Several benzoquinone ansamycins [10], as well as a number of small molecule, purine- and pyrazole-based synthetic inhibitor compounds based on the interactions of RAD [11-15], are now being evaluated for cancer treatment.

The first Hsp90 inhibitors to be analysed in detail were the benzoquinone ansamycins. As a result, the first identifications of Hsp90 inhibitor resistance involved these agents. As outlined below (Section 2), these pioneering studies uncovered mechanisms of resistance that are now thought to affect just these benzoquinone ansamycin inhibitors, not the synthetic purine- and pyrazole-based drugs designed around the interactions of RAD. It is now apparent that a factor that is much more significant in Hsp90 drug resistance is the potent ability of N-domain inhibitors to induce the heat shock response, a response that increases the cellular levels of prosurvival chaperones (Section 3 below). In addition, several types of cancer cell are able to evade apoptosis when treated with these drugs through mechanisms that are much more subtle (Section 6 below). First though, since resistance that is acquired through mutation of the drug target still remains one of the most serious causes of the failure of cancer chemotherapy, we consider whether an appreciable resistance to N-domain inhibitors could arise by the mutation, or altered modification, of Hsp90 itself.

## 2. Resistance by Mutation or Modification of the Drug Target—Hsp90

**Figure 1.**
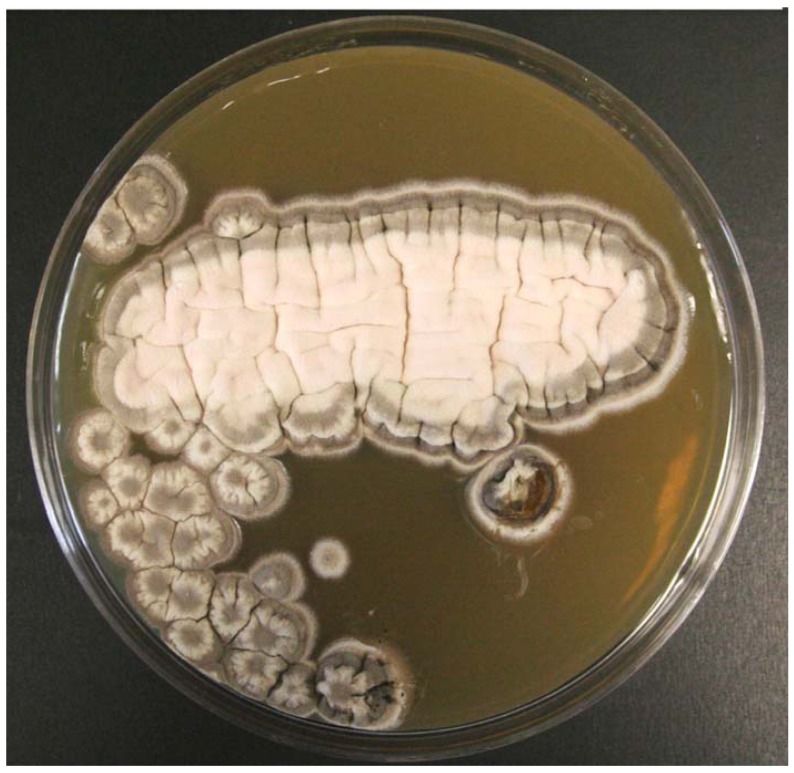
A petri dish culture of the mycopathogenic fungus *Humicola fuscoatra*, one of the several soil fungi that produce RAD. Others are to be found in the rhizosphere of plants, where the production of this Hsp90-targetting antibiotic may help facilitate the establishment of the fungal-plant symbiotic relationship [18].

One potential advantage of an inhibitor that binds within the highly-conserved ADP/ATP binding pocket of Hsp90 is the expectation that resistance should not readily arise through a mutation that weakens drug binding. Most mutations within this pocket should severely compromise the function of Hsp90, since they would involve changes to the amino acid residues that facilitate the ATP binding, the ATP-mediated conformational changes or ATPase steps of the Hsp90 chaperone cycle. Should nature have evolved drug-resistant forms of Hsp90, these would be expected to occur in the organisms that make Hsp90-targetting antibiotics so as to protect these microbes against their antibiotic production. We therefore recently investigated the Hsp90 family protein (HtpG) of the organism that makes GdA, *S. hygroscopicus* [16]; also the Hsp90 of a fungus that produces RAD (*Humicola fuscoatra*; Figure 1) [17].

GdA binds almost all Hsp90 family proteins with high selectivity. To date, only the HtpG of *S. hygroscopicus* [16] and the Hsp90 of the nematode *Caenorhabditis elegans* [19] have been reported not to bind this antibiotic. *S. hygroscopicus* HtpG is altered in a number of the residues forming interactions with GdA in cocrystal structures of this antibiotic bound to the yeast and human Hsp90s [20,21]. Two of these changes, involving the loss of charged amino acid side chains on one face of the ATP binding pocket, generated partial GdA resistance when introduced into the Hsp90 of a model eukaryotic cell, the yeast *Saccharomyces cerevisiae.* Inserted into the Hsp90 of the latter organism these mutations (E88G and N92L in combination) generated a ∼10-fold weaker affinity for GdA *in vitro* and 2.5-fold increases in IC(50) for GdA and 17-allylaminodemethoxygeldanamycin (17-AAG) inhibition of growth *in vivo* [16]. The crystal structure of GdA in complex with this E88G, N92L double mutant form of yeast Hsp90 revealed these changes were weakening the interactions of the chaperone with the C-12 methoxy group of GdA [16]. Exactly why the *C. elegans* Hsp90 lacks GdA binding has yet to be established. Unlike the *S. hygroscopicus* HtpG, this *C. elegans* chaperone is not altered in any of the amino acids normally interacting with GdA. Furthermore, when expressed heterologously as the sole Hsp90 of yeast, *C. elegans* Hsp90 renders cells more susceptible—not resistant—to *in vivo* inhibition by GdA [22].

Investigating the cytosolic Hsp90 of *H. fuscoatra* (Figure 1), we discovered that it had an unusually low affinity for RAD, but not GdA [17]. *H. fuscoatra* Hsp90 is largely unaltered in the residues forming direct, or water molecule/Mg^2+^ ion-bridged, interactions with RAD in cocrystal structures [21]. Its only unusual feature is an isoleucine (I33), rather than the normal leucine, as the residue immediately following the glutamate (E32) that catalyses the intrinsic ATPase reaction of Hsp90. Introduced into the Hsp90 of yeast this single, conservative L to I amino acid change reproduced the weakened *in vitro* binding of RAD displayed by the Hsp90 of *H. fuscoatra* and rendered cells partially resistant to RAD *in vivo* [17]. The crystal structure of RAD in complex with this L34I mutant yeast Hsp90 revealed that this conservative change was causing an increased hydration in the vicinity of the bound RAD molecule [17].

Only partial—not complete—resistance to either GdA or RAD has been generated in these studies by introducing into the Hsp90 of yeast cells the unusual features of the N-domain ADP/ATP binding site of Hsp90s from those organisms that make Hsp90-targetting antibiotics. Therefore this work would appear to partly validate the prediction that most changes to the amino acid residues that facilitate N-domain inhibitor binding would compromise the essential chaperone function of Hsp90. The *S. hygroscopicus* HtpG—though resistant to GdA—still has normal affinities for RAD and for two other inhibitors (NVP-AUY922 and VER49009) whose binding is based on the interactions of RAD [16]. Conversely the *H. fuscoatra* Hsp90-though partially resistant to RAD—still has a normal affinity for GdA [17]. This is an indication that, should Hsp90 mutations such as these ever cause a degree of drug resistance in the clinic, it should be possible to overcome such resistance by switching from an Hsp90 inhibitor drug based on the interactions of GdA to one based on the interactions of RAD, or *vice versa*.

Other work has shown that resistance to N-domain inhibitors can arise as a consequence of mutations in human Hsp90 close to, but not immediately part of, the conserved residues that make up the active site (e.g., I123T or A121N in Hsp90α and I128T or A116N in Hsp90β [23,24]). These mutations are thought to influence the relative stability of different conformations, or cochaperone associations, of Hsp90. Potentially these latter mutations might pose a more serious problem in the clinic and confer a degree of resistance to all inhibitor classes of Hsp90. Moreover, it is known that alterations to the posttranslational modification status of Hsp90 can *sensitise* cells to N-domain inhibitors [25-27]. Whether altered phosphorylation status of Hsp90 is a potential route to increased resistance is still unproven, but it is a possibility suggested by a recent study [28]. The acetylation status of Hsp90 might also be important. Inhibition of histone deacetylase 6 (HDAC6) causes a deacetylation and disrupted chaperone function of Hsp90 [25]. An aberrant acetylation of Hsp90, due to upregulation of histone deacetylases and down-regulation of histone acetyltransferase (HAT), has also been noted as one of the consequences of resistance to the Bcr-abl tyrosine kinase inhibitor Imatinib [29]. The degree to which drug resistance in human malignancies may be affected by the relative stability of the different conformations, the cochaperone associations, or the posttranslational modifications of Hsp90 urgently needs further investigation.

## 3. Systems of Resistance Apparently Selective for the Benzoquinone Ansamycins, not the Synthetic Purine- and Pyrazole-Based Hsp90 Inhibitors

### 3.1. Resistance to Benzoquinone Ansamycins through the Overexpression of Drug Efflux Pumps

Resistance to two benzoquinone ansamycins (GdA and herbimycin A) was noted in human breast tumour cells even before the target of these inhibitors was identified as Hsp90, linked to the multidrug resistance (MDR) phenotype and the overexpression of P-glycoprotein (P-gp) [30]. Since then, a number of studies have shown resistance to 17-AAG in cells overexpressing P-gp and/or the related MRP-1 efflux pump. Importantly, such 17-AAG resistant cells still remain sensitive to the synthetic purine- and pyrazole-based inhibitors of Hsp90, since the latter are not P-gp substrates [15,31,32]. Thus, tumours such as adrenocortical carcinoma that naturally overexpress P-gp and exhibit appreciable resistance to 17-AAG are still susceptible to synthetic Hsp90 inhibitors such as BIIB021 [31].

### 3.2. Resistance to Benzoquinone Ansamycins through Lowered Expression of NQO1

**Scheme 1.**
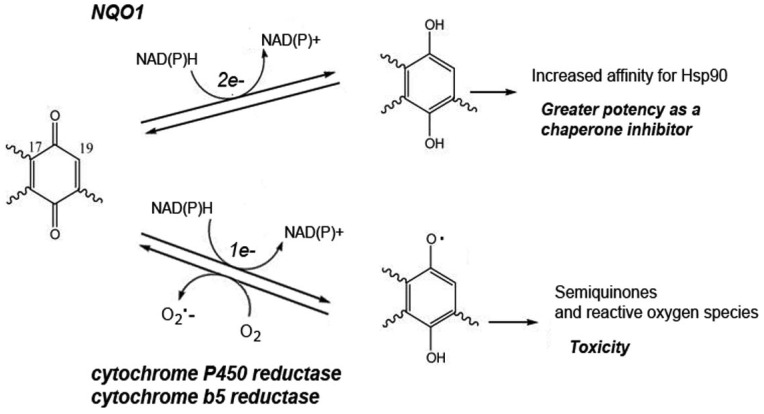
A two electron reduction of benzoquinone ansamycins by NQO1 generates the hydroquinone. In contrast a one electron reduction generates the unstable semiquinone, allowing the formation of the superoxide radical (O_2_•^−^) by redox cycling.

Resistance to benzoquinone ansamycins, but not the synthetic purine- and pyrazole-based N-domain inhibitors, can also be apparent with a lowered activity of the quinone-metabolizing enzyme NAD(P)H: quinone oxidoreductase 1 (NQO1) [32-36]. NQO1 catalyses the reduction of benzoquinone ansamycins to the hydroquinone (Scheme 1), a reduced state which they bind Hsp90 more tightly and are therefore a more potent inhibitors of this chaperone. Exposing glioblastoma cells in culture to progressively higher levels of 17-AAG, cells with an elevated resistance to this inhibitor were selected [36]. Their acquired 17-AAG resistance could be directly attributed to their lowered NQO1 activity, as it was abrogated by the NQO1 inhibitor ES936 [36].

Doubts have been expressed as to whether NQO1 activity constitutes an important factor in resistance to the hydroquinone form of 17-AAG (IPI-504) in the clinic. Analyzing both the potency of IPI-504, the hydroquinone/quinone (HQ/Q) ratio of this drug and NQO1 enzyme abundance in 30 cancer cell lines, levels of NQO1 expression were found to be correlated with the intracellular HQ/Q ratio of IPI-504 in only a subset of these cell lines and were, overall, poorly correlated with the growth inhibitory effects of IPI-504 [37].

Besides a reduction by NQO1 (an obligate two-electron reductase), the benzoquinone ansamycins can also undergo a reduction by one-electron reductases such as NADPH cytochrome P450 reductase and NADH cytochrome b5 reductase. The latter activities are particularly abundant in the liver. Such one-electron reduction generates the semiquinone radical, the instability of which then causes redox cycling with the production of superoxide (O_2_•^−^; Scheme 1).

Hepatotoxicity of the benzoquinone ansamycins *in vivo* is often attributed to the oxidative stress resulting from this superoxide formation, the greater hepatoxicity of GdA as compared to 17-AAG being attributed to a greater propensity of the former compound to undergo a reduction by one-electron reductases [38]. Recently though, another possible explanation of the toxicity of benzoquinone ansamycins has been suggested—inhibition of mitochondrial function through the benzoquinone moiety interacting, in a non Hsp90-dependent manner, with the mitochondrial permeability transition (MPT) pore [39]. Whatever the basis of the toxic effects of the benzoquinone, these should be eliminated using the nonquinone derivatives of macbecin and of GdA which are now under development [40,41].

### 3.3. A Possible Resistance through a Reaction of Benzoquinone Ansamycins with Thiols

The possibility of a glutathione (GSH)-based mechanism of resistance to the benzoquinone ansamycins was indicated by the finding that cells are sensitized to 17-AAG by treatment with buthionine sulfoximine, an inhibitor of GSH synthesis [42]. As the oxidation state of the cellular GSH pool is a major determinant of cellular redox balance, this sensitization might reflect a lack of reduction of 17-AAG to its more potent hydroquinone state in the prooxidant intracellular environment of buthionine sulfoximine-treated cells. It might also reflect an altered redox regulation of the heat shock response [43] in these cells. Alternatively, since benzoquinone ansamycins can form GSH adducts at the 19 position of the quinone ring, it might reflect a detoxification through the covalent attachment of GSH [38]. The 17-AAG resistance conferred by Hsp27 up-regulation has been in part attributed to its effects on the cellular pool of GSH [42].

## 4. Resistance Caused by N-Domain Inhibitors Activating the Heat Shock Response

Activation of the heat shock response is now recognized as one of the most important causes of acquired resistance to N-domain inhibitors. Hsp90 is a negative regulator of heat shock transcription factor 1 (HSF1), such that its inhibition leads to the formation of the active HSF-1 homotrimers needed for the induction of the heat shock response [44,45]. At least in the yeast model system, a point mutation in Hsp90 is capable of rendering HSF-1 constitutively active [46].

Blocking the actions of HSF-1 is now a hot topic in cancer biology due to the fact that malignant cells have a high dependency upon the activity of HSF-1 [47]. Cancer cells show a much greater dependence on HSF-1 for proliferation and survival as compared to their nontransformed counterparts, a reflection of their need for high chaperone levels [48]. Eliminating the function of HSF-1 in mice is also protective against oncogenic mutations *in vivo* [48].

Chaperones that are subject to a strong induction by HSF-1 (notably Hsp70 and Hsp27) have major prosurvival functions, acting to inhibit cytochrome c and TNF-mediated cell death (see Section 7 below) [42,49-52]. A number of studies have now shown that the upregulation of these chaperones leads to marked increases in the cellular capacity for evasion of apoptosis. Resistance to 17-AAG-induced apoptosis is apparent with the overexpression of Hsp70 in human AML HL-60 cells [53]. The high endogenous levels of Hsp70 in Bcr-Abl-expressing CML-BC K562 cells are an important factor in the 17-AAG resistance of these cells, since resistance is abrogated by transfection of a small interfering RNA (siRNA) to Hsp70 [53]. A similar silencing of Hsp70 expression promotes proteasome-dependent degradation of Hsp90 client proteins, G1 cell-cycle arrest, and extensive tumour-specific apoptosis in other human cancer cell lines [50]. Furthermore, even in cells rendered resistant to GdA and 17-AAG by P-gp overexpression, silencing of Hsp27 and/or Hsp70 decreases the IC(50) for 17-AAG 10-fold as compared to the control transfected cells [51]. Evidently therefore the ability of 17-AAG to induce the heat shock response is a much more important factor than P-gp overexpression in acquired resistance to 17-AAG.

### 4.1. Combination Drug Therapy Can Potentially Overcome this Detrimental Effect of N-Domain Inhibitors Inducing the Heat Shock Response

This induction of the heat shock response appears to be an unfortunate drawback of all the N-domain inhibitors of Hsp90, including the newer purine and 4,5-diaryisoxazole resorcinol compounds [14,51,54]. However it would appear that it can be avoided by switching to a recently-developed class of Hsp90 inhibitor that binds with high-affinity to the C-terminal region of the chaperone. One of these C-terminal inhibitors, KU135, has been shown to promote client degradation, but not HSF-1 induction, and to act as a potent inducer of mitochondria-mediated apoptosis [55].

Alternatively, it should be possible to overcome the detrimental effects of N-domain inhibitors inducing HSF-1 by using these inhibitors in combination with other drugs. In this respect inhibitors of the Hsp70 family of molecular chaperones are potentially of tremendous potential. The status of Hsp70 drug development has recently been reviewed [52,56], the targeting of Hsp72 by one such compound enhancing Hsp90 inhibitor-induced apoptosis in myeloma cells [57]. In cell culture a combinatorial use of 17-AAG and cisplatin has also shown promise, the cisplatin strongly suppressing the HSF-1 activation by 17-AAG [54]. These two agents act synergistically, leading to an increased apoptosis as compared to the use of each agent alone [54]. Furthermore, since HDAC6 histone deacetylase is required for HSF-1 activation [58], there is the prospect that Hsp90 inhibitors might act synergistically with HDAC inhibitors in promoting the growth arrest and apoptosis of tumour cells.

Small molecule inhibitors that act directly on HSF-1 have also been identified, including the natural flavonoid quercitin [59,60] and the benzylidene lactam compound KNK437 [61]. Cotreatment of human AML HL-60 cells with 17-AAG and KNK437 attenuates the 17-AAG-mediated induction of Hsp70 and increases levels of 17-AAG-induced apoptosis [53].

### 4.2. The Potential Problems of Abrogating HSF-1 Activity

Inactivating HSF-1 has the potential drawback that it may act to the detriment of normal tissue function. In counteracting age-related functional decline, also certain diseases of protein homeostasis, the usual aim would be to activate, rather than inactivate, HSF-1. Drugs that act as activators, rather than inhibitors of HSF-1 are a potential therapy for—with proven ability to suppress—protein misfolding/aggregation and associated toxicity in model systems of amyotrophic lateral sclerosis, Parkinson's and polyQ expansion-associated disease [62-64]. Furthermore, HSF-1 tends to lose its stress inducibility as part of the process of normal ageing, possibly a significant factor in age-related functional decline [65]. 17-AAG, probably through its ability to stimulate HSF-1 and thereby enhance chaperone expression, promotes a recovery from contraction-induced damage in the skeletal muscles of aged mice [66].

N-domain inhibitors of Hsp90 are just some of the many known small molecule activators of HSF-1. Others include protein synthesis inhibitors (e.g., puromycin), amino acid analogues (e.g., azetidine and canavanine), proteasome inhibitors (e.g., MG132 and lactacystin), and serine protease inhibitors (e.g., N-tosyl-L-phenylalanine chloromethyl ketone and N-p-tosyl-L-lysine chloromethyl ketone) [67,68]. Other inducers of HSF-1 activity include phospholipase A2, arachidonate and the antiproliferative prostaglandins that are mediators of the inflammatory process [68]. Celastrol, a quinone methide triterpene derived from a traditional Chinese medicinal herb, has been studied both as an HSF-1 activator [43] and as an inhibitor of the Hsp90 system (the cochaperone p23 [69] and of Hsp90/Cdc37 interaction [70]). Furthermore, while the objective in cancer treatment might be to suppress the activity of HSF-1 [48], one should probably not view all activators of HSF-1 as being detrimental in this respect. For example, proteasome inhibitors are activators of HSF-1, yet one (bortezomib) has already been approved for the treatment of multiple myeloma since it operates as a potent enhancer of TRAIL-induced apoptosis [71].

## 5. Cochaperone Levels are a Probable Influence over Hsp90 Inhibitor Resistance

Hsp90 function is regulated by a number of accessory proteins or “cochaperones”. These regulate Hsp90 by modulating its rate of ATP hydrolysis (Aha1, Cdc37, p23), its conformational flexibility (p23, Sgt1), the binding of specific substrates or the assembly of higher-order protein complexes (Hop, Cdc37, Sgt1), or the ubiquitination of client proteins [C-terminus of Hsp70-interacting protein (CHIP)]. These cochaperones have diverse effects on the biochemical activities of Hsp90. It is not unreasonable therefore to expect that some of them will impact on drug resistance. Loss or downregulation of a number of these cochaperones has already been shown to render cells hypersensitive to N-domain inhibitors [72-74]. Furthermore, like Hsp90 itself, these cochaperones are often upregulated in cancer and some—though not all—are heat shock proteins, subject to an induction with the activation of HSF-1.

One cochaperone that is attracting interest is Cdc37—important for the presentation of nascent protein kinases to the Hsp90 system. With many oncogenic protein kinases dependent on its activity, Cdc37 is itself a potential drug target [75,76]. Cdc37 levels are elevated in prostate cancer and pre-malignant prostatic neoplasias, Cdc37 overexpression in the prostate epithelium of transgenic mice having been found to result in a high incidence of hyperplasia [77]. In still other transgenic mice the overexpression of Cdc37 was shown to promote mammary tumour formation, collaborating with c-myc in the transformation of multiple tissues [78]. Furthermore a dominant negative mutant form of Cdc37 that lacks the Hsp90 binding domain was found to block proliferation and promote apoptosis [79]. But does Cdc37 influence resistance to inhibitors of Hsp90? Structural studies reveal that Cdc37 binds at the mouth of the ATP binding pocket of Hsp90 [80], in a way that would be expected to interfere with the dissociation of inhibitor molecules bound within this pocket. It might thereby increase the apparent affinity of drugs for Hsp90 (as observed in tumours [1-3]). In yeast, the *CDC37* gene displays haploinsufficiency with regard to RAD resistance (our unpublished observations). There are therefore some indirect indications that the levels of Cdc37 protein might be a significant factor in resistance to N-domain inhibitors of Hsp90.

Other cochaperones that may influence Hsp90 drug resistance include Aha1—potentially important in the enhanced ATPase activity of tumour cell Hsp90 [73]—and p23—a protein essential for perinatal survival [81]. In cell culture, appreciable resistance to N-domain inhibitors can arise with the reinforced Aha1 binding that results from a mutation in the cytosolic Hsp90 [23]. High levels of p23 appear to promote increased metastases and drug resistance in breast cancer, without affecting the oestrogen-dependent proliferative response [82]. Conversely, depletion of p23 enhances apoptotic cell death in response to endoplasmic reticulum stress [83], this apoptosis being reflected in a caspase-dependent cleavage in the C-terminal tail of p23 [84]. A similar cleavage of p23 is also seen in chemotherapy-induced apoptosis [85].

## 6. Resistance May Arise from an Altered Susceptibility of a Key Client Protein to Proteasomal Degradation

In response to N-domain inhibitor treatment most oncogenic clients of Hsp90 are destabilized through the concerted actions of ubiquitin ligases and the proteasomal pathway. The co-chaperone/E3 ubiquitin ligase CHIP is instrumental in these events [86]. However, in certain specific instances, a key Hsp90 client seems not to be destabilized rapidly when Hsp90 is inhibited. This may, in turn, impact on sensitivity to Hsp90 inhibitors. Notably N-domain inhibitor treatment has been shown to transiently activate certain protein kinases (PKR [87], Erb2B [88] and src [89]).

The controlling factor may be the association of the Hsp90 client with a cell type-specific cofactor, the latter a component that influences whether the client is destabilized or protected from degradation. Hypoxia-inducible factor-1alpha (HIF1α), an Hsp90 client important for the angiogenesis of tumour growth, is partly protected from Hsp90 inhibitor-mediated degradation in COS cells when—in the form of a heterodimer with aryl hydrocarbon receptor nuclear translocator (ARNT)—it constitutes a functional transcription factor [90]. This capacity of ARNT to both up-regulate the activity of HIF1α as well as diminish the sensitivity of HIF1α to destabilisation by GdA reflects its ability to compete for the Hsp90 binding site on HIF1α [90]. HIF1α upregulation is important in the survival and angiogenic activity of radioresistant lung cancer cells, a situation where Hsp90 inhibitor treatment suppresses the HIF1α/Hsp90 interaction and HIF1α expression [91]. The promise and the potential problems of targeting Hsp90 in tumour vascularisation has recently been reviewed [9].

The polyubiquitination and subsequent proteasomal degradation of this HIF1α, as well as that of another key Hsp90 client, ErbB2, involves the Cullin5 E3 ubiquitin ligase [92]. Stabilization of HIF1α under hypoxia is achieved through the impairment of this HIF1α degradation process [93]. Furthermore the expression of Cullin 5 is decreased in many breast cancers suggesting that its levels could conceivably become limiting for ErbB2 degradation [9]. The p53 tumour suppressor, a key regulator of apoptosis, is yet another situation where the protection of a client against the actions of an E3 ubiquitin ligase (in this case Mdm2) appear to impact on drug sensitivity. Unlike wild type p53, mutant forms of p53 are normally stable and inhibitory to Mdm2. This may be the reason why 17-AAG induces a stronger viability loss in cancer cells that contain these mutant, oncogenic p53s than in cells containing the wild-type, tumour suppressor form of p53 [94].

Another aspect, often overlooked, is due consideration of whether the activity of the proteasome might itself be appreciably affected by Hsp90 inhibitor treatment. GdA and 17-AAG are known to cause a partial disruption of 26 S proteasome structure, a reflection of a requirement for Hsp90 in the assembly and structural maintenance of the 20S regulatory particle (or “cap”) of the proteasome [95].

## 7. Resistance to N-Domain Inhibitors through an Inability to Promote Apoptosis

Multiple stresses or developmental cues can provoke a cell to undergo apoptosis; either by the intrinsic or mitochondrial death pathway, or by the extrinsic or receptor-mediated death pathway. These pathways—intrinsic or extrinsic—are linked through the Bcl-2 family of proteins. The latter include both pro- (e.g., Bax) and anti-apoptotic (e.g., Bcl-XL) members; able to promote or inhibit apoptosis respectively through their direct action on the mitochondrial permeability transition (MPT) pore. Bax and/or Bak form the pore, while Bcl-2, Bcl-XL or Mcl-1 inhibits its formation. Generally apoptosis occurs in response to the release of mitochondrial cytochrome c, which then nucleates the Apaf-1/caspase-9 apoptosome [28]. The apoptosome cleaves pro-caspase-9 to its active form of caspase-9, which in turn activates the effector caspase-3.

There are many ways in which cells can avoid apoptosis. This can involve reducing the expression of proapoptotic factors (e.g., p53 or death receptor ligands such as TNF or TRAIL); increasing the expression of antiapoptotic factors (e.g., Bcl2, Raf, Hsp70), or activating inhibitor of apoptosis proteins (IAPs) such as survivin. Alternatively it can reflect altered activity of the oncogenic tyrosine kinases that act to inhibit apoptosome assembly (see below).

### 7.1. Depending on the Cell Type, There can be a Number of Reasons why N-Domain Inhibitors do not always Promote Apoptosis

Since chaperones generally promote cell survival, most of their actions are antiapoptotic (reviewed in [49]). Hsp90 is antiapoptotic in many of its effects, primarily through its actions in promoting the NF-κB mediated inhibition of apoptosis and in blocking certain steps of the apoptotic cascades (Table 1). There are a number of reports of either Hsp90 inhibition (e.g., [55,96,97]) or siRNA knockdown of Hsp90 [98] inducing apoptosis. Counteracting this, however, are the effects of N-domain inhibitors causing the HSF-1-directed induction of prosurvival chaperones (Hsp70, Hsp27). Notably, the Hsp70 binding to Bax inhibits the 17-AAG-mediated mitochondrial localization of Bax and mitochondria-initiated events of apoptosis [53]. However increases in Hsp70 are not the only reason why N-domain inhibitor treatment may not always promote apoptosis.

A number of studies focus on survivin, an Hsp90 client IAP virtually undetectable in most normal tissues, but highly expressed in a range of human tumours, where its presence is often correlated with both accelerated relapse and chemotherapy resistance. Survivin release from mitochondria results in the inhibition of caspase-9 activation and a block to apoptosis. As survivin is an Hsp90 client, its depletion is often followed in analyzing Hsp90 inhibitor effects [32]. However, others have found the opposite effect (GdA and 17-AAG causing an overexpression of survivin and an enhancement of cell survival in certain cancer cell lines [109]).

The p53 tumour suppressor is yet another paradoxical situation. Here the mutant, oncogenic form of p53 appears to sensitise cells to 17-AAG [94]. In normal cells there is a tight control over wild-type p53 level, acting mainly through the Mdm2 E3 ubiquitin ligase. This control is lost in tumours harbouring a mutant p53 which exhibit a dramatic, constitutive hyperstabilization of p53—both mutant and wild type forms—due to a stable complex between Hsp90 and the mutant p53 inhibiting Mdm2 and CHIP [94]. 17-AAG disaggregates this complex, inducing a stronger viability loss in cancer cells containing mutant p53 than those containing the wild-type p53 [94].

In endothelial cells and vascular endothelial growth factor (VEGF) receptor-positive primary leukemias, resistance to GdA-induced apoptosis is due to VEGF inducing the antiapoptotic Bcl-2 [110]. This overexpression of Bcl2 is associated with an Hsp90-dependent stabilization of HIF1α under hypoxia [93]. Remarkably however, while Bcl2 overexpression reduces cell killing by 17-AAG, it is reported not to affect killing by the synthetic Hsp90 inhibitor BIIB021 [31].

Oncogenic tyrosine kinases frequently act to inhibit assembly of the Apaf-1/caspase-9 apotosome, allowing binding of Hsp90 to Apaf-1 to prevent cytochrome c-induced recruitment of caspase 9 to Apaf-1 [28]. This Hsp90-apoptosome interaction may contribute to chemoresistance in leukemias and would appear to reflect Hsp90β phosphorylation at Ser 226/Ser 255. In untransformed cells, where Hsp90β is only weakly associated with the apoptosome, expression of the nonphosphorylatable S226A/S255A mutant Hsp90β conferred resistance to cytochrome c-induced apoptosome activation. In contrast, in Bcr-Abl-positive mouse bone marrow cells, an expression of the same S226A/S255A mutant Hsp90β conferred resistance to Imatinib, a Bcr-abl tyrosine kinase inhibitor used in CML [28]. Resistance to tyrosine kinase inhibitors such as Imatinib through mutation of the kinase itself is a major problem that can potentially be overcome by Hsp90 inhibition [111].

From these few specific examples, one must conclude that there is almost certainly a high degree of cell selectivity in whether an N-domain inhibitor of Hsp90 can efficiently promote apoptosis, also in the key steps in apoptotic cascades responsive to Hsp90 inhibition and Hsp70 level.

### 7.2. Resistance to N-Domain Inhibitors through Inaccessibility of the Drug to the Mitochondrial Hsp90

In resistance to diverse cancer drugs (not just Hsp90 inhibitors) much attention now focuses on the Hsp90 proteins of the mitochondrion. Importantly Hsp90, also the mitochondrial Hsp90 family protein TRAP1 (Hsp75), are abundant in both the intermembrane space and matrix of the mitochondria of tumour cells, but often either undetectable or expressed at very low levels in the mitochondria of most normal tissues [112]. Furthermore, an association is frequently found between the MDR phenotype and the overexpression of TRAP1. TRAP1 levels are markedly elevated in HT-29 colorectal carcinoma cells resistant to 5-fluorouracil, oxaliplatin and irinotecan; while TRAP1 overexpression can recreate this MDR phenotype in a number of different neoplastic cell types [113]. In contrast an RNAi knockdown of either TRAP1 and/or the TRAP1 interactor protein Sorcin was found to sensitize colon cancer cells to cancer agents that induce apoptosis, suggesting that agents targeting TRAP1/Sorcin (e.g., Shepherdin, Gamitrinibs, see below) may constitute a therapeutic strategy for colorectal tumours [114].

These effects appear to reflect an antiapoptotic role of the Hsp90 chaperones of tumour cell mitochondria, whereby these chaperones act to suppress the MPT [112]. In the mitochondrial matrix Hsp90 and TRAP1 bind the matrix immunophilin, cyclophilin D (CypD). CypD is a key component of the organized MPT pore, whose transition to an open conductance state can lead to mitochondrial swelling and cell death through apoptosis or necrosis. Consistent with TRAP1 suppressing apoptosis, siRNA knockdown of TRAP-1 was shown to trigger a CypD-dependent apoptosis while, conversely, TRAP1 overexpression was protective against oxidative stress-induced apoptosis [112].

The conventional N-domain inhibitors of Hsp90 appear not to accumulate in tumour mitochondria [6]. Therefore, in drug trials, they may have failed to inhibit the mitochondrial pools of Hsp90 and TRAP1. To overcome this problem, small molecule inhibitors have been designed that will selectively accumulate in the mitochondria of tumour cells and target the Hsp90/TRAP1 of these organelles (e.g., shepherdin, a peptidomimetic inhibitor of the interaction between Hsp90 and its survivin client; and gamitrinibs, linking 17-AAG to lipophilic cations that act as mitochondrial targeting moieties) [6]. By inhibiting the mitochondrial Hsp90 of tumours these mitochondria-directed Hsp90 antagonists should trigger an opening of the MPT pore and a CypD-dependent apoptotic cell death. Normal cells should, it has been suggested, be relatively insensitive to these drugs as their mitochondria are substantially devoid of Hsp90 chaperones and use alternative mechanisms for CypD regulation of the MPT pore [6].

Certain Hsp90 inhibitors may not need to access the mitochondrial matrix in order to act on the MPT. The benzoquinone moiety of GdA and 17-AAG has been found to interact, in a non Hsp90-dependent manner, with the outer membrane voltage dependent anion channel (VDAC)[39]. As VDAC is a regulator of MPT, this binding of benzoquinone ansamycins (but not synthetic purine- and pyrazole-based Hsp90 inhibitors) may collapse the mitochondrial membrane potential by a direct VDAC-mediated effect on the MPT pore [39].

## 8. Conclusions

Malignant cells are subject to a number of factors that threaten their survival, not just external stressors such as hypoxia and nutrient deprivation, but also internal stresses such as the accumulation of mutated and partially folded proteins, and the inappropriate activity of deregulated signalling pathways. They are, as a result, addicted to high levels of activity of Hsp90 and Hsp70 family chaperones, also the HSF-1 transcription factor that is a key regulator of Hsp90 and Hsp70 expression. Hsp90, Hsp70 and HSF-1 are all promising targets for anticancer drug development [47,48,52,56], yet it is inhibitors of Hsp90 that have been propelled to the fore as chaperone inhibitors. This reflects the high selectivity of these agents for their primary target (Hsp90), the multiple oncogenic clients of Hsp90 (e.g., Akt, mutant p53, ErbB2) that are secondary targets, and the greater sensitivity of transformed as compared to normal cells to these drugs.

It is now apparent that cancers will display variable levels of resistance to these Hsp90 inhibitors, both intrinsic and acquired. Cell culture studies have now provided firm genetic evidence of some of the routes to such resistance (summarized in Table 2). More challenging is achieving a full picture of the diverse factors that can prevent N-domain inhibitors causing apoptosis. Here a number of cellular characteristics—many of them specific to each target cell—must be taken into account (e.g., the native levels of chaperones, including Hsp70; whether the signaling in this cell is “primed” for apoptosis through the mitochondrial, or the receptor-mediated death pathway; the proapoptotic versus antiapoptotic activities in this cell type that are dependent on Hsp90 (Table 1; Section 7.1.); and whether the Hsp90 drug is able to access all of these various activities (Section 7.2.). Furthermore, it might also be necessary to consider the effects of Hsp90 inhibition on the epigenetic control of gene expression [115]. In many cancer cell types, only a systems approach will provide a complete picture and identify the key activities that should be targeted by any chaperone-directed drug therapy.

A separate, yet equally challenging task, is achieving a full appreciation of how Hsp90 inhibition will impact on resistance to other drugs. Already there are a number of studies showing that N-domain inhibitors of Hsp90 can act synergistically with certain of the other drugs approved for chemotherapy, potentiating the effects of the latter (e.g., [54,116,117]). It is possible therefore that, in the longer term, Hsp90 drugs will find their most widespread usage in combination drug therapy. Moreover, recent work in fungal cells has shown that, at levels subinhibitory for growth, N-domain inhibitors of Hsp90 will block the emergence of acquired resistance to other drugs [118]. This raises the issue of whether a similar, long term exposure of early stage cancer cells to low, noncytotoxic levels of Hsp90 inhibitor might suppress the hypermutator phenotype of cancer cells, thereby arresting the progression to more aggressive tumour stages and the emergence of MDR. Orally available Hsp90 inhibitors are currently being evaluated [119], although it is unclear whether a long term treatment with such drugs would be an option in therapy. Despite this, it will be fascinating to know the longer-term effects of dampening Hsp90 activity on tumour progression and on acquired drug resistance.

## Figures and Tables

**Table 1.** Some of the reported involvements of Hsp90 in apoptosis.

***Antiapoptotic roles of Hsp90***	
Stabilisation and activation of Akt serine/threonine kinase [99].	Activates the protein kinase that destabilises the IκB inhibitor subunit of NF-κB, thereby allowing NF-κB to translocate into the nucleus and activate cell survival genes [100]. Induces phosphorylation of Bad, the resultant Bad dissociation from Bcl-XL preventing apoptosis [101]. Inhibits Jnk-mediated cell death, by catalyzing phosphorylation and inactivation of the Jnk activator ASK-1 [102]. Necessary for maintenance of telomerase activity [103].

Inhibition of apoptosome formation	Hsp90 binding to cytosolic Apaf-1 inhibits the formation of the active Apaf-1/caspase-9 apoptosome in response to release of cytochrome c from mitochondria [104].

Stabilisation of death-associated protein kinase-1, a calcium/calmodulin (CaM)-regulated serine/threonine kinase [105].	May, in some situations, be a resistance factor to TNF-α-induced cell death [106].

Stabilisation of survivin, a protein highly expressed in a range of human tumours but not normal differentiated cells [107].	Inhibits apoptosis by inhibiting caspases—survivin release from mitochondria during apoptosis causing an inhibition of caspase-9 activation. Promotes cancer cell growth by stabilizing microtubules during mitosis.

***Proapoptotic roles of Hsp90***	

Interaction with hypoxia-responsive HGTD-P, a proapoptotic protein which transmits hypoxic signals directly to mitochondria [108].	Essential for HGTD-P to be translocated into mitochondria and induce the mitochondrial death pathway.

Stabilisation of death-associated protein kinase-1 [105]	Participates in cell death in response to various cytokine signals [106].

Stabilisation of p53	Stable complex formation between Hsp90 and mutant p53 interferes with normal p53 function by inhibiting Mdm2 and CHIP [94].

**Table 2.** Summary of the firmly established mechanisms of N-domain inhibitor resistance.

**Mechanism**	**Comments**	**Relevance for human malignancies**
Mutation, possibly also by an altered posttranslational modification, of Hsp90 itself (see Section 2.).	Yeast molecular genetics and mammalian cell culture studies reveal that a degree of resistance to N-domain inhibitors can arise this way.	No evidence of resistance arising this way to date.

MDR, P-gp overexpression (see Section 3.1.).	Only relevant for the benzoquinone inhibitors, since the synthetic purine- and pyrazole-based inhibitors of Hsp90 are not P-gp substrates [15,31,32].	MDR is important in several malignancies. However its effects should be overridden by the use of synthetic purine- and pyrazole-based Hsp90 inhibitors.

Lowered Expression of NQO1 (see Section 3.2.).	Only relevant for the benzoquinone inhibitors.	Uncertain.

Induction of the heat shock response (see Section 4.).	Relevant for all the N-domain inhibitors of Hsp90; due to these drugs being potent inducers of HSF-1 activity, and thereby increasing the level of prosurvival chaperones (see Sections 4.1. and 7.1.).	There are good indications indications that it may be possible to overcome this unfortunate drawback by the appropriate combinatorial drug treatment (see Section 4.2.).
